# Damage evaluation and precursor of sandstone under the uniaxial compression: Insights from the strain-field heterogeneity

**DOI:** 10.1371/journal.pone.0262054

**Published:** 2021-12-29

**Authors:** Hongming Cheng, Xiaobin Yang, Zewen Zhang, Wenlong Li, Zhangxuan Ning

**Affiliations:** 1 School of Emergency Management and Safety Engineering, China University of Mining and Technology-Beijing, Beijing, China; 2 School of Coal Engineering, Shanxi Datong University, Datong, China; Sapienza University of Rome: Universita degli Studi di Roma La Sapienza, ITALY

## Abstract

The stress-induced microcrack evolution in rock specimens causes a series of physical changes and heterogeneous deformations. Some of these attributes (such as sound, electricity, heat, etc.) have been effectively used to identify the damage state and precursory information of the rock specimens. However, the strain-field heterogeneity has not been investigated previously. In this study, the relationship of the strain-field heterogeneity and damage evolution of three sandstone specimens under the uniaxial compressive load was analyzed statistically. The acoustic emission (AE) and two-dimensional digital image correlation were employed for real-time evaluation of the AE parameters and strain-field heterogeneity. The results showed that the strain-field heterogeneity was closely related to the rock damage that amplified with the applied stress, and exhibited two features; numerical difference and spatial concentration. Subsequently, these two features were characterized by the two proposed heterogeneous quantitative indicators (i.e., the degree and space heterogeneities). Further, their four transition processes were in agreement with the damage stages confirmed by AE parameters: a relatively constant trend; growth with a relatively constant rate; drastic increase trend; and increase with a high rate to maximum value. Moreover, a time sequence chain for damage precursor was built, where the heterogeneous quantitative indicators and AE parameters differed in sensitivity to microcrack development and can be used as a damage warning at the varying magnitude of the external load.

## Introduction

Rock damage and catastrophic failure are the key issues in rock engineering, and often cause accidents, such as landslide, rock burst, roof collapse, and dynamic disaster in coal mines [1–4]. Rock damage mechanism and fracture process have been widely investigated [4–6]. However, damage state assessment and precursory information identification remain difficult and have attracted significant attention of researchers. The rock damage process is characterized by the inner microcrack closure, initiation, penetration, and coalescence [7–9], accompanied by variations in physical features (such as sound, electricity, heat, etc.) that are associated with damage evolution in rocks, Browning *et al*. used acoustic emissions (AE) evaluate the onset and evolution of new crack damage caused by inelastic processes in rocks under both conventional and true triaxial loading conditions [10]; Kim *et al*. quantified damage evolution in granite using various methods including crack volumetric strain, b value, damage parameter from the moment tensor, and AE energy, and pointed out AE energy method was more attractive with regard to the practical applicability and reliability [11]; Heap *et al*. used AE (micro-seismicity) to evaluate further crack damage in cycle loading [12]; Eccles *et al*. found the precursory electric potential signals prior to failure in both saturated and dry samples of the quartz-rich sandstones [13]; Pandey and Chand indicated the change in temperature at any point/zone of the material indicates the deformation and damage [14]. Generally, the macroscopic stress-strain curve and stress/strain field response of the damage evolution in rocks are considerably evident, and Zhang *et al*. [15] believed that the stress/strain measurement in time and space is essential for analyzing the damage mechanism and fracture process. For instance, E. Eberhardt *et al*. [16] divided the entire stress-strain curve into five important stages corresponding to the characteristic stress (i.e., crack closure (*σ*_*cc*_), crack initiation (*σ*_*ci*_), crack damage (*σ*_*cd*_), peak (*σ*_*p*_), and residual (*σ*_*c*_) stresses). Subsequently, various researchers investigated volumetric strain, crack volumetric strain, lateral strain, and lateral strain response methods to estimate the stages of stress-strain curve and characteristic stress [17–20]. In stress/strain field, through the laboratory-based experimental techniques, such as rock surface observation, thin-section viewing, and X-ray imaging, it was observed that the heterogeneous micro-structure controlled the damage and fracture initiation in rocks, and the direct manifestation was the heterogeneity of stress/strain field. However, measurement of the temporal and spatial distribution of the stress/strain field heterogeneity through these conventional experiments was difficult [21–23]. Previously, numerical methods were employed to visually study the influence of micro-structure heterogeneity on rock damage; it was observed that the micro-structure heterogeneity induced significantly different local stresses compared to the entire stress field, which created a strain concentration zone resulting in a potential damage initiation [24, 25]. However, the micro-structures considered in physical models were simple and incapable to completely describe the stress/strain heterogeneity. Therefore, unconventional experiment-based techniques were required for studying the stress/strain heterogeneity in rock damage. Thus, a non-contact technique, digital image correlation (DIC), was employed to obtain complete strain-field in rock damage with external stress, and observed to be feasible to investigate stress-induced strain heterogeneity in compression and tensile tests [26–30]. Through this technique, existing research related to the phenomenon of the heterogeneous strain-field have indicated that the rock catastrophic failure began with the strain localization, which appears close to the rock peak stress or in the pre-peak plastic stage [28, 29, 31, 32]. Further, to quantify the damage precursory information from strain-field, Ma *et al*. [33] calculated the variance of shear strain-field using statistical method, and the abrupt change in variance was considered as the rock fracture precursor; Zhang *et al*. [34] considered the obvious growth of the differentiation rate in strain-field as the rock fracture precursor. Similarly, Shirole *et al*. [35] used the standard deviation to study multi-scale strain-field heterogeneity, and reported that the evolution of strain-field heterogeneity and damage were interconnected.

Moreover, various precursors during rock damage have common features in generation mechanism, and the acquisition and appearance of the heterogeneous strain-field are more convenient, direct, and comprehensive than the physical features in identifying the damage state and precursory information [36–38]. To the best of authors knowledge, the existing literature lacks in quantitatively evaluating the formation and development process of the strain-field heterogeneity. Further, some deficiencies in expressing the rock damage process and damage precursor from the heterogeneous strain-field still exist, and the difference between the strain-field and physical features, such as the acoustic emission (AE), has not been investigated previously in time sequence. Therefore, in this study, experiments were conducted on red sandstone under uniaxial loading in sync with AE and two-dimensional (2D)-DIC measurements to track the strain-field and AE signal in real time during rock damage evolution. Moreover, the heterogeneous strain-field was quantitatively characterized in reference to the stages of rock damage by proposing the space and degree statistical indicators. Additionally, the generation mechanism of the heterogeneous strain-field and AE signal was analyzed to establish the time sequence chain by the heterogeneous statistical indicators and AE parameters. Subsequently, the precursory information in the time sequence chain was used to identify various levels of damage in the rock specimens.

## Experimental program

**Fig 1:**
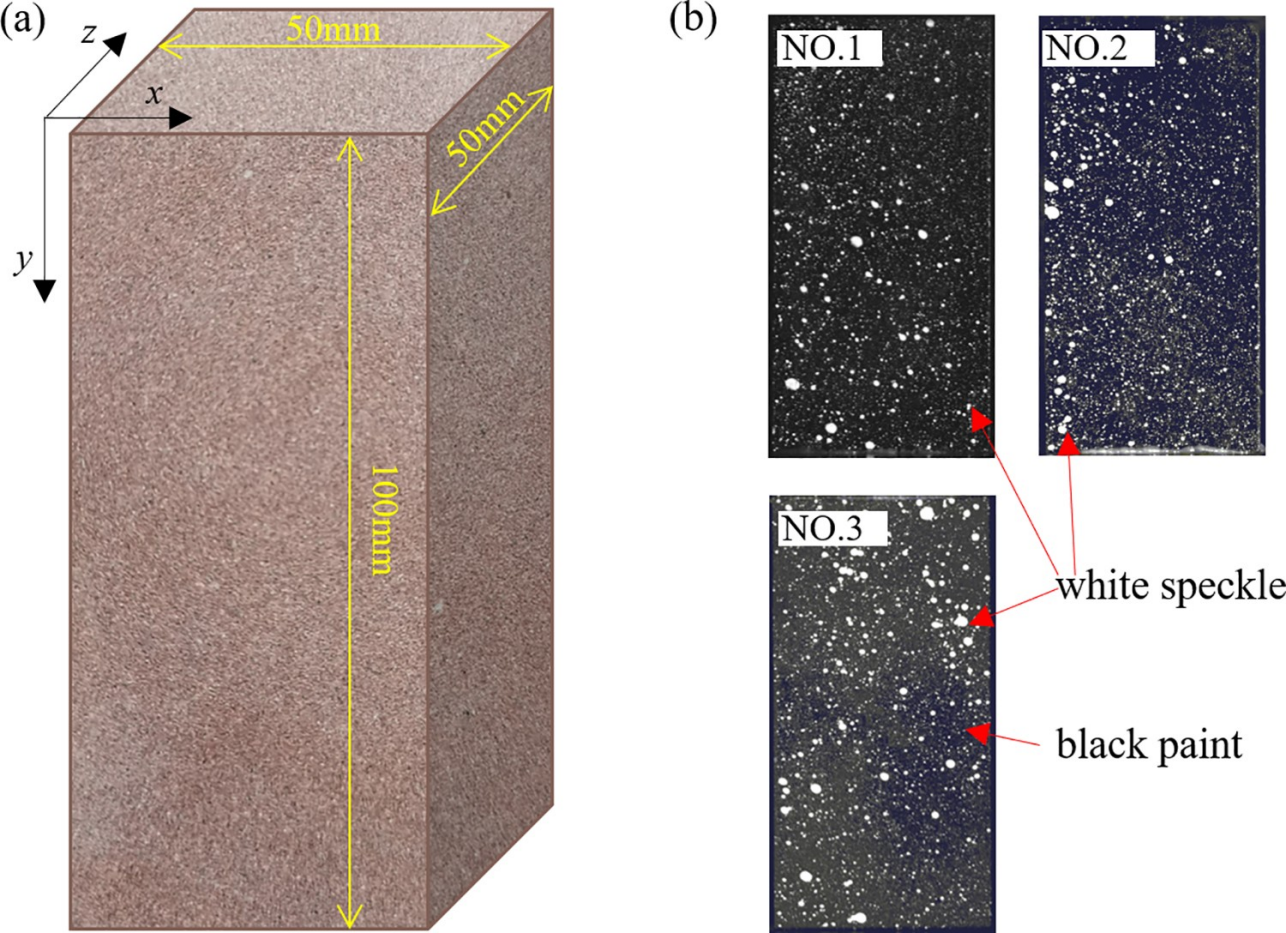
Geometric dimension and speckle field, (a) geometric dimension of specimens; (b) speckle field of the specimens No. 1, No. 2, and No. 3.

In the present study, red sandstone having a density of 2.43 g/cm^3^ was employed for the experiments owing to its homogeneous and isotropic nature. The red sandstone, possessing a uniform and fine texture with negligible visible joints, comprised quartz (63.5%), calcite (14.3%), feldspar (12.4%), clay (5.5%), and other minerals (4.3%). Parallelepiped of dimensions 50 × 50× 100 mm were prepared as testing specimens by cutting and grinding (refer Fig 1(A), y-direction is loading direction), and the parallelism of all faces of the specimens was controlled within ± 0.02 mm. Six specimens with smaller anisotropy were selected through acoustic wave test in different directions. Before the test, the artificial speckle field, as shown in Fig 1(B), was created on the selected specimens’ surface, and its production process: all faces of the specimen were smoothed and cleaned firstly, and the x-y plane (Fig 1(A)) was selected to spray a layer of black paint as the background of the speckle field secondly, finally, a small amount of white paint was randomly sprayed on the surface when the black paint dried.

**Fig 2:**
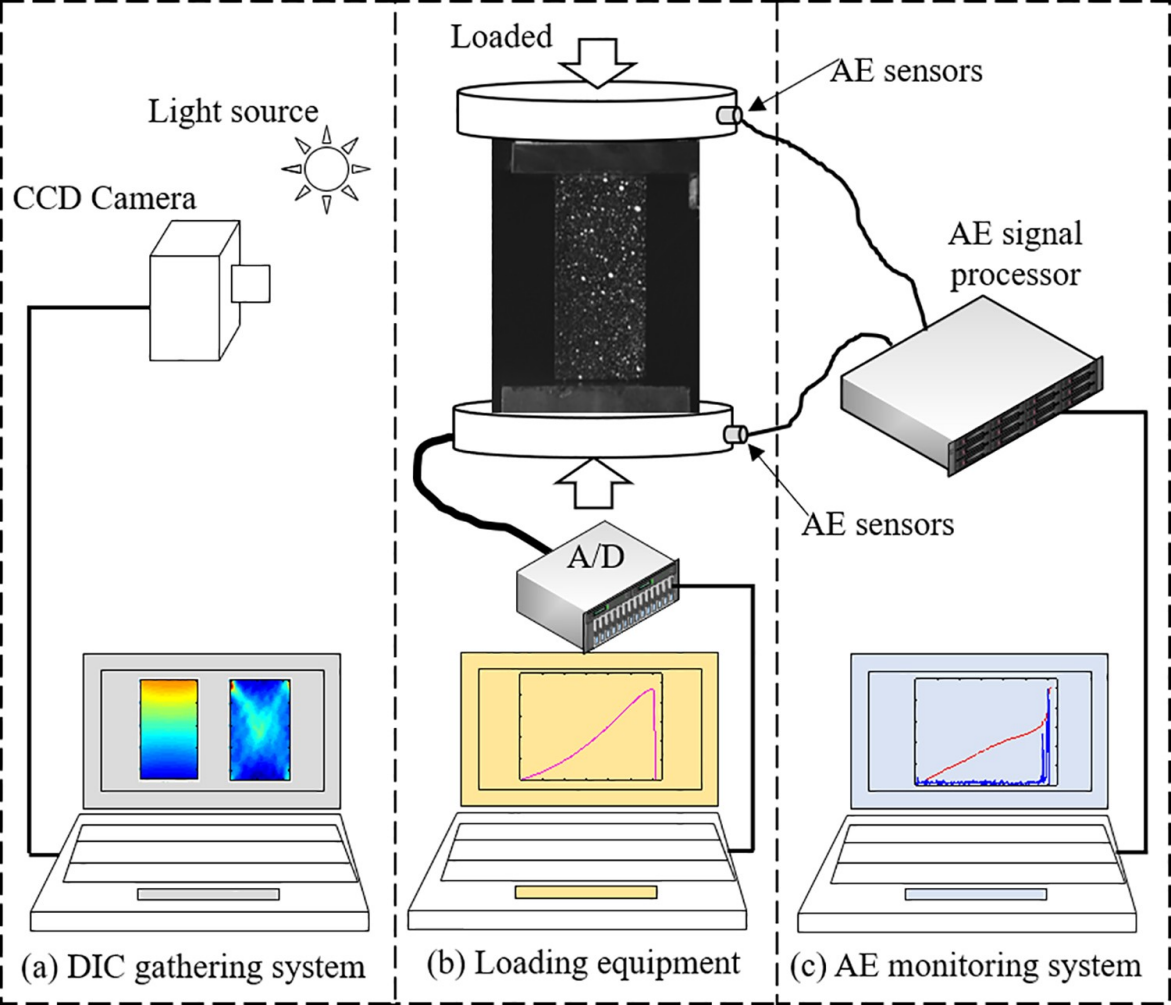
Layout of loading equipment and monitoring systems.

Fig 2 shows the uniaxial compression experimental system consisted of loading equipment, AE monitoring, and DIC gathering systems. The RLJW-2000 electro-hydraulic servo loading machine, which can record load, displacement, and loading time, was employed in this study. The AE monitoring system (manufactured by Vallen Systeme, Germany) recorded the AE parameters (e.g., ring, accumulative ring, energy, etc.) through AE sensors during rock deformation and damage process. Two AE sensors were arranged at an angle of 150° on the loading head to prevent being damaged by the collapse of rock specimens. The AE monitoring system preamplifier gain, threshold value, and sampling rate were set to 40 dB, 50 dB, and 10 MHz, respectively. The DIC gathering system comprising a Charge Coupled Device (CCD) industrial camera, lens (the focal length of 50 mm or 25 mm), and cold light source was used to capture speckle images of specimen surface, with a rate, graphic resolution, and object plane resolution of 10 frames per second, 2048 × 2048 pixel, and 0.07 mm/pixel, respectively.

First, a pre-load of 10 kN was applied at a rate of 0.5 kN/s to stabilize the rock specimens. Then, the load and displacement of the loading system were reset, and the displacement-controlled axial loading rate was set to 0.005 mm/s. Subsequently, the sampling rate and store path of the AE monitoring and DIC gathering systems were adjusted. Finally, the loading equipment, DIC gathering, and AE monitoring systems were triggered simultaneously, and the experimental data were collected throughout the entire loading process.

## Experimental results

The stress, AE parameters, and speckle images of six specimens in time sequence were obtained experimentally, while the artificial speckle fell out at some point/zone of the specimens during loading, and finally three specimens with complete surface speckle images were considered in this study, and marked as specimen No. 1, No. 2, and No. 3 (Fig 1).

### Damage evolution process in rock specimens

**Fig 3:**
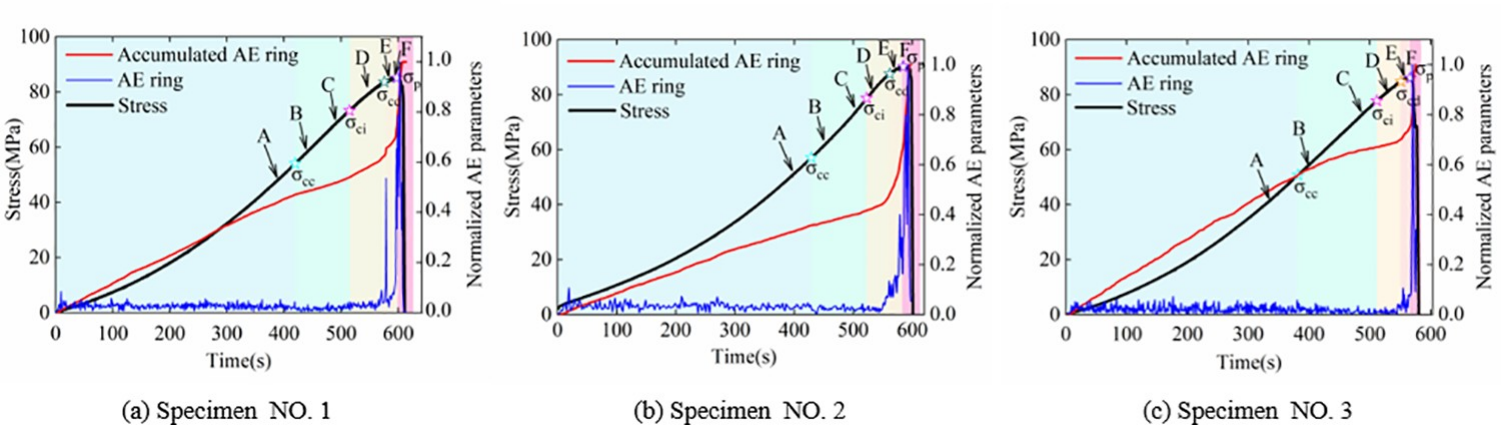
Time sequence evolution curves of stress and normalized AE parameters for the three specimens.

The normalized AE and accumulated AE rings and stress-time curves of the three specimens are shown in Fig 3. The AE parameter variational characteristics have been used to identify the damage evolution state for rock specimens in various existing studies [39–41]. Fig 3 shows five evolution stages of the stress: Crack closure stage (*0–σ*_*cc*_); linear elastic strain stage (*σ*_*cc*_*–σ*_*ci*_); crack stable (*σ*_*ci*_*–σ*_*cd*_) and unstable (*σ*_*cd*_*–σ*_*p*_) growth stages (sometimes combined and called plastic strain stage); and post-peak stage (*σ*_*p*_*–0*).

For all three specimens at the crack closure stage, due to the initial microcrack closure in rock specimens, there were few high-frequency elastic waves emitting from new microcrack, and the AE ring showed a very slight variation with increasing external stress, while the accumulated AE ring increased linearly, and the stress-time curve showed concave growth. The crack closure stresses (*σ*_*cc*_) of the three specimens were 54.0, 57.1 and 50.7 MPa, respectively. At linear elastic strain stage, the AE ring showed a very slight decreasing trend with low fluctuation in amplitude, and the accumulated AE ring also increased gradually, and the stress-time curve increased linearly. The crack initiation stresses (*σ*_*ci*_) were 73.4, 78.7 and 77.8 MPa for the three specimens, respectively. The microcrack in rock specimens initiated at crack stable growth stage, and the growth trend of the AE and accumulated AE rings increased. When the cracks developed to a certain extent, i.e., entering the rock unstable growth stage, the AE ring exhibited the first sudden rise (usually used as a precursor for rock rupture), and accumulated AE ring showed an accelerated growth. The crack damage stresses (*σ*_*cd*_) for the three specimens were 85.5, 90.3 and 86.3 MPa, respectively. At the post-peak stage (often sustaining very short time in uniaxial compression test), owing to the crack coalescence, the AE ring appeared densely and the accumulated AE ring reached the maximum value.

### Evolution characteristics of strain-field

**Fig 4:**
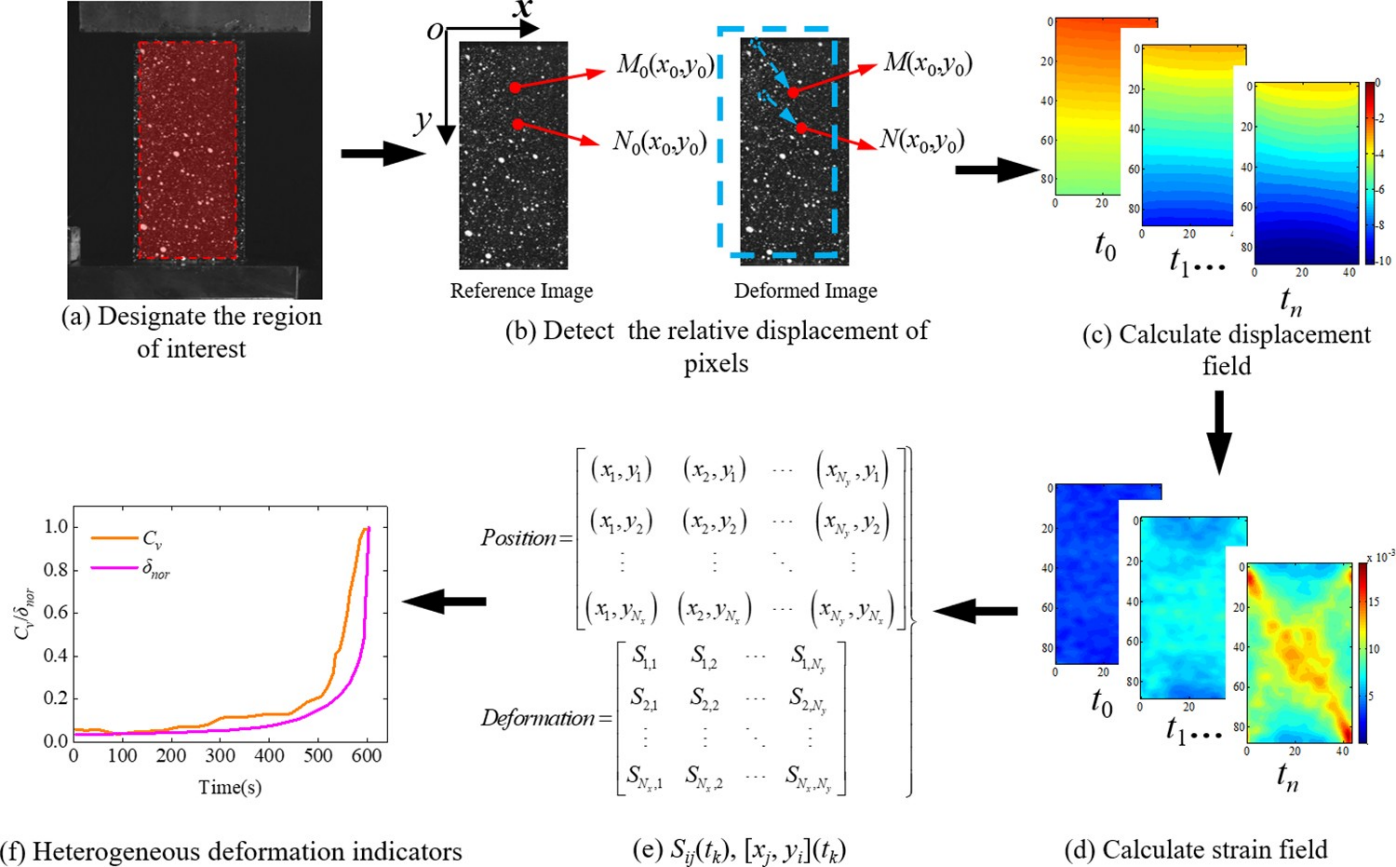
Workflow of 2D-DIC to obtain heterogeneous deformation indicators.

**Fig 5:**
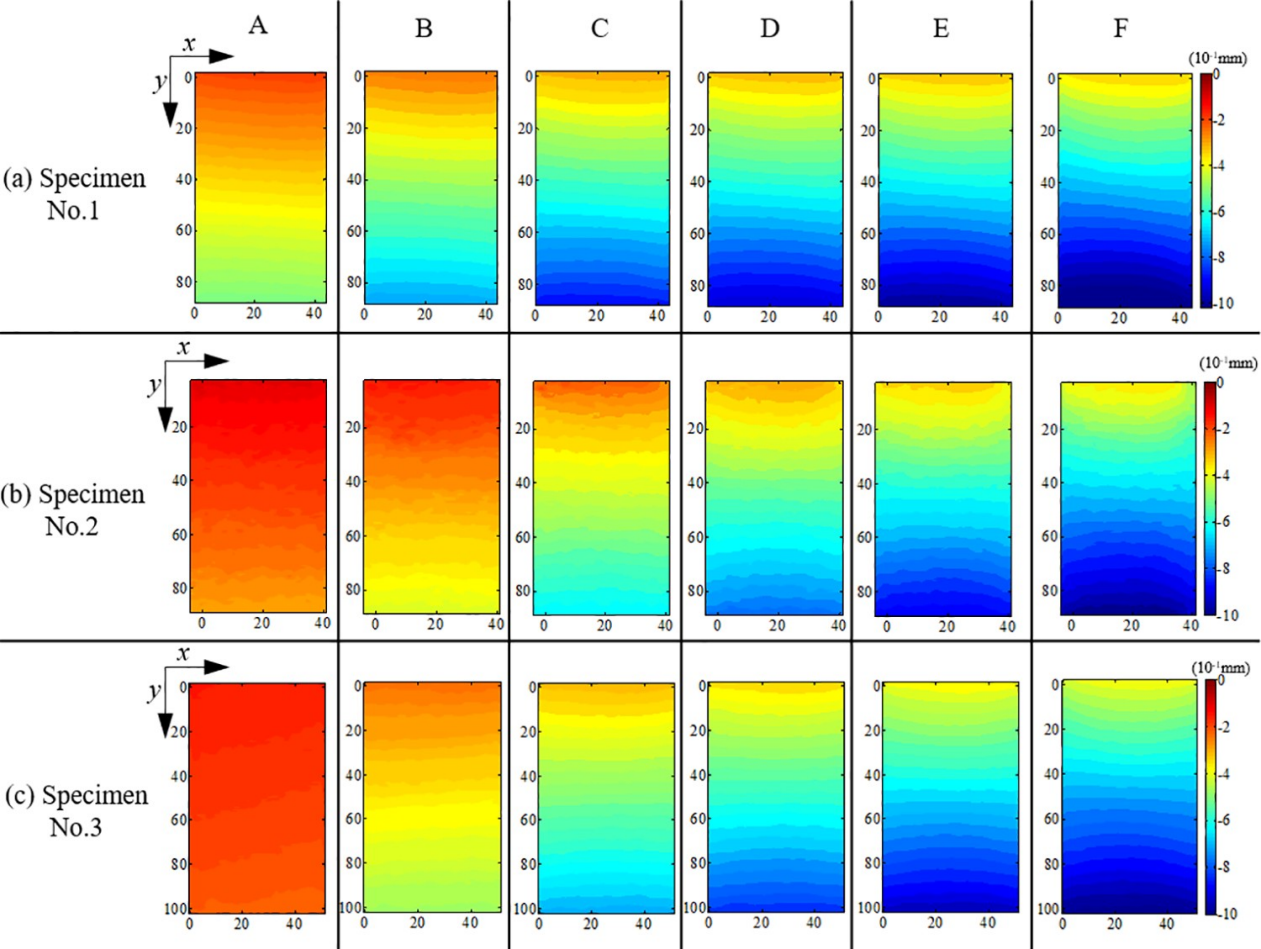
Vertical displacement-field (*u*_*y*_) for (a) Specimen No. 1; (b) Specimen No. 2; (c) Specimen No. 3.

**Fig 6:**
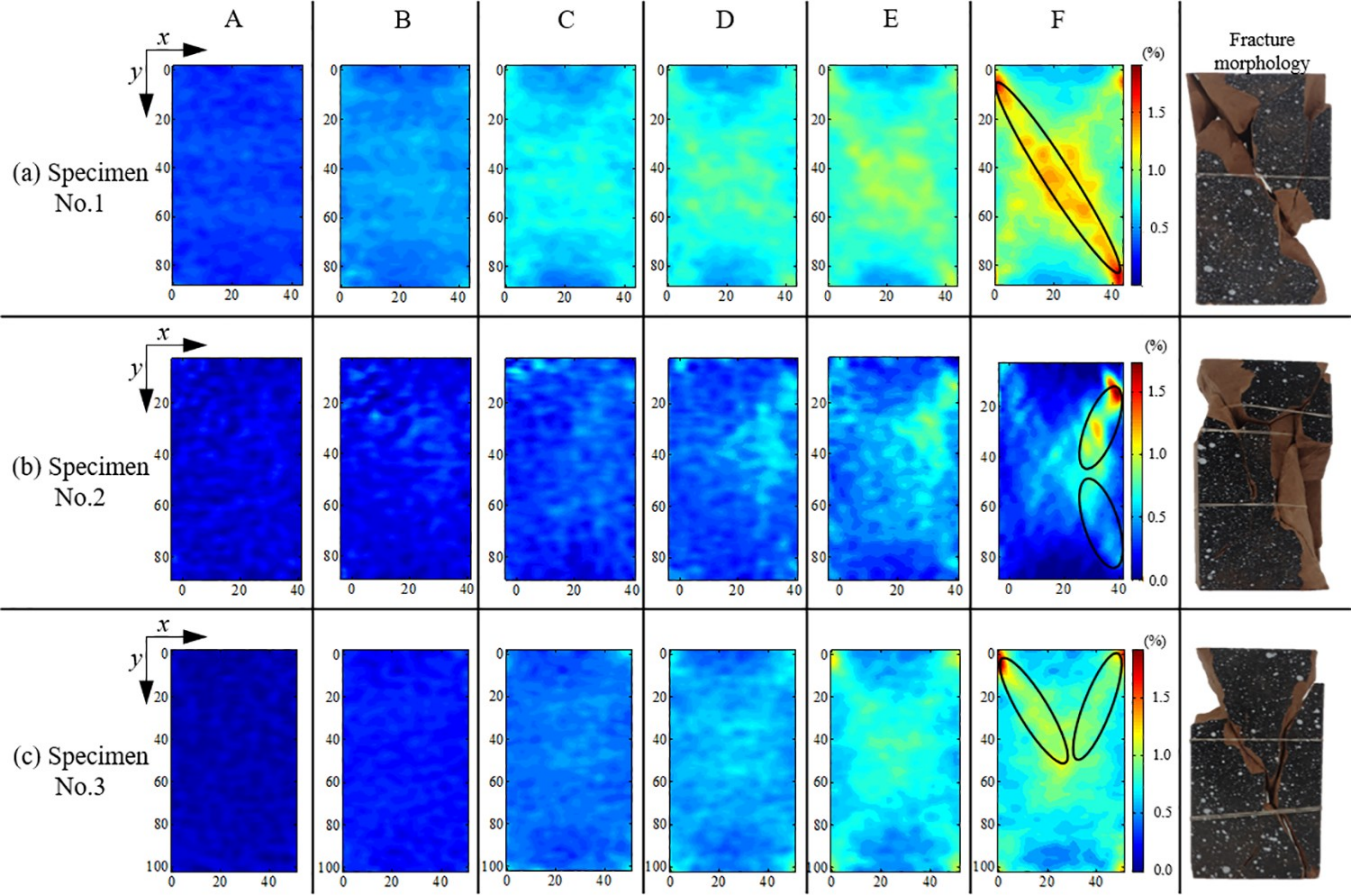
Shear strain-field (*γ*_xy_) and fracture morphology for (a) Specimen No. 1; (b) Specimen No. 2; (c) Specimen No. 3.

The evolution characteristics of surface strain-field in the rock specimens are produced by the inherent microcracking. In this study, six points (recorded as markers A, B, C, D, and F) in each stress-time curve of the three specimens were selected to obtain the evolution characteristics of the strain-field in time sequence, as shown in Fig 3. Marker A was located at crack closure stage; markers B and C were at pre and post linear elastic strain stages, respectively; markers D and E were at crack stable and unstable growth stages, respectively; marker F was located at peak stress. The selected speckle images were processed through the software of the 2D-DIC and its workflow is shown in Fig 4(A)–4(D): the reference image and the region of interest (ROI) is specified initially, then the ROI of the reference image is partitioned a number of subsets, and each subset must be uniquely identifiable. Finally, the calculation of displacement field is to track the subsets (initially defined in the ROI of the reference) in the deformed images, and the strain field is calculated based on the continuum mechanics from the displacement field. The vertical displacement-field (*u*_*y*_, along the loading direction) and shear strain-field (*γ*_*xy*_) of the specimens No. 1, No. 2, and No. 3 are shown in Figs 5 and 6, respectively.

In Fig 5(A)–5(C), the *u*_*y*_ contours shown are for markers A, B, C, D, E, F from left to right, and the *x* and *y* axes represent width and length of the ROI. Fig 5 shows that *u*_*y*_ at the bottom is greater than that at the top for all specimens due to application of load from bottom of each specimen. At marker A, the vertical displacement in specimen surface varied slightly without evident change in gradient, specifically in specimens No. 2 and No. 3. At markers B and C, with an increase in load, each part of the specimen developed vertical displacement uniformly with an evident displacement gradient. Once the inherent heterogeneous micro-structure (generally considered as the new microcrack) started developing at plastic strain stage (markers D and E), the displacement gradient changed unevenly in the horizontal direction for all three specimens. For instance, the displacement gradient of *u*_*y*_ at the top and bottom of the specimens at marker F showed concave and convex trends, respectively. The heterogeneous variation in the displacement-field contributed in expanding and connecting the microcracks; this phenomenon was more evident for *γ*_xy_.

In Fig 6(A)–6(C), the images are *γ*_*xy*_ contours for markers A, B, C, D, E, F and fracture morphology from left to right, and the *x* and *y* axes represent width and length of the ROI. As shown in Fig 6, the strain value in *γ*_xy_ at marker A is relatively small, and larger strain points are distributed sporadically (due to low stress and microcrack closure). At initial linear elastic strain stage (marker B), the strain was relatively uniform throughout the entire specimen due to small microcrack initiation in the three specimens. Moreover, *γ*_xy_ does not show a clear difference between the larger and smaller strain points. However, later at this stage (marker C), a little microcrack was developed, and a gathering zone of the larger strain points was appeared, which could be the latent damage initiation. When the plastic strain occurred, owing to the sufficient microcrack development and rearrangement, the strain localization was appeared, i.e., the regional difference between the larger and smaller strain points formed for the three specimens (refer marker D in Fig 6). After continued loading, the regional difference developed continuously and rapidly (markers E and F in Fig 6), and a single band was observed for specimen No. 1 (refer ellipse at marker F in Fig 6(A)), while two bands developed with an angle for specimens No. 2 and No. 3 (refer ellipse at marker F in Fig 6(B) and 6(C), respectively). Finally, for all three specimens, the strain of the regional difference reached a certain degree where the macroscopic fracture was occurred (refer broken rock specimens in Fig 6). In general, the evolution of *γ*_xy_ was heterogeneous with increasing external stress, and the strain-field heterogeneity showed two evident features: numerical difference and spatial concentration.

### Quantitative indicators of the strain-field heterogeneity

In the statistical method, the strain-field information, obtained at a certain time (*t*_*k*_), was considered as a sample, and a series of *N*_*x*_ × *N*_*y*_ matrices with 2D-DIC were obtained including displacement and strain. It represents the temporo-spatial evolution of strain behaviors, where *N*_*x*_ and *N*_*y*_ indicate the number of rows and columns of the matrices, respectively. Let *S*_*ij*_(*t*_*k*_), [*x*_*j*_, *y*_*i*_](*t*_*k*_) be the matrix element representing the strain and position, respectively, and corresponding to the *ij*-th pixel at time *t*_*k*_ (*i* and *j* are the row and column indices, respectively, *i* = 1, 2,…,*N*_*x*_, *j* = 1, 2,…, *N*_*y*_), as shown in Fig 4(E).

As shown in Fig 4(B), the plane-coordinate system was set up on the upper left corner of the ROI, where x and y axes represent width and length of the ROI, respectively. It is evident that each pixel has a definite position in experimental surface of the rock specimen. The rows and columns of the matrices were converted to coordinate value as follows:

$$\left[x_{j},y_{i}]\left(t_{k}\right)=[j\frac{W}{PX},i\frac{L}{PY}\right]$$ (1)

where *W* and *L* are width and length of the ROI, respectively; *PX* and *PY* denote number of pixel points on the transverse and longitudinal axes of the ROI, respectively.

Therefore, the numerical difference of strain-field heterogeneity can be described through statistics indicators, such as average, variance, standard deviation, and variation coefficient. Further, the spatial concentration of the strain-field heterogeneity can be quantified through spatial correlation coefficient. In this study, the normalization standard deviation of the strain-field (termed as degree heterogeneity (*δ*_*nor*_)) and spatial correlation coefficient of the larger strain points in the strain-field (termed as space heterogeneity (*C*_*v*_)) were proposed to characterize the numerical difference and spatial concentration of the strain-field heterogeneity, respectively. The standard deviation (*δ*) and *δ*_*nor*_ of the strain-field at time *t*_*k*_ were calculated using Eqs (2) and (3), respectively. In Eq (2), ${\bar{S}}_{ij}(t_{k})$, which is the mean value of the entire strain-field at time *t*_*k*_, was calculated using Eq (4). Finally, *δ*_*nor*_ were plotted in time sequence, as shown in Fig 4(F). The standard deviation of the strain-field heterogeneity in rock specimens have been investigated with loading previously, which experiences three stages and reaches the maximum value (*δ*_*max*_) at peak stress [31, 35, 42]. This implies that *δ*_*nor*_ ranges between 0 and 1 at pre-peak stress stage, and the strain-field heterogeneity increases with the increasing *δ*_*nor*_.

$$\delta (t_{k})=\sqrt{\frac{1}{N_{x}N_{y}}\overset{N_{x}}{\underset{j=1}{\sum }}\overset{N_{y}}{\underset{i=1}{\sum }}{\left(S_{ij}(t_{k})-{\bar{S}}_{ij}(t_{k})\right)}^{2}}$$ (2)

$$\delta_{nor}(t_{k})=\frac{\delta_{nor}(t_{k})}{\delta_{\max}}$$ (3)

where

$${\bar{S}}_{ij}\left(t_{k}\right)=\frac{1}{N_{x}N_{y}}\overset{N_{x}}{\underset{j=1}{\sum }}\overset{N_{y}}{\underset{i=1}{\sum }}S_{ij}\left(t_{k}\right)$$ (4)

Before calculating *C*_*v*_, the range of larger strain points in strain-field need to be selected. Referring to the existing studies of the strain localization on rock specimen surface [31, 43, 44], the top 10% of larger strain points in strain-field were considered to determine *C*_*v*_ as expressed in Eq (5). In Eq (5), $\bar{x(t_{k})}$ and $\bar{y(t_{k})}$, which are the mean value of the position coordinates of the larger strain points at time *t*_*k*_, was calculated using Eqs (6) and (7). The *C*_*v*_-time curve is shown in Fig 4(F). In spatial statistics, the spatial correlation coefficient was used to evaluate the correlation of points in space, and the maximum value was 1 in the case of linear dependence, which decreased for other cases. Therefore, the value of *C*_*v*_ ranged between 0 and 1 and varied with the degree of concentration.

$$C_{v}(t_{k})=\frac{\left|\sum_{i=1}^{n}(x_{i}(t_{k})-\bar{x(t_{k})})(y_{i}(t_{k})-\bar{y(t_{k})})\right|}{\sqrt{\sum_{i=1}^{n}{\left(x_{i}(t_{k})-\bar{x(t_{k})}\right)}^{2}}\sqrt{\sum_{i=1}^{n}{\left(y_{i}(t_{k})-\bar{y(t_{k})}\right)}^{2}}}$$ (5)

where

$$\bar{x(t_{k})}=\frac{1}{n}\overset{n}{\underset{i}{\sum }}x_{i}(t_{k})$$ (6)

$$\bar{y(t_{k})}=\frac{1}{n}\overset{n}{\underset{i}{\sum }}y_{i}(t_{k})$$ (7)

### Evolution of the heterogeneous quantitative indicators

**Fig 7:**
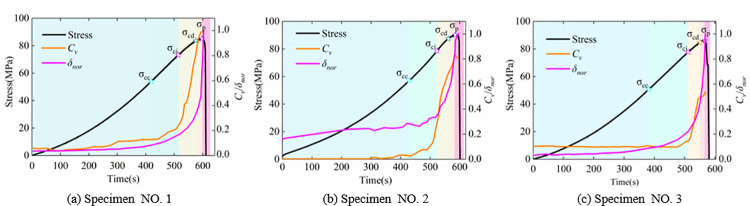
Variation in *δ*_nor_ and *C*_*v*_ with loading for the three specimens.

From the aforementioned equations, *δ*_*nor*_ and *C*_*v*_ of *γ*_xy_ for specimens No. 1, No. 2, and No. 3 were calculated. Fig 7 shows variation of *δ*_*nor*_ and *C*_*v*_ with loading for the three specimens. Although *δ*_*nor*_ and *C*_*v*_ were not similar to the stress, *δ*_*nor*_ and *C*_*v*_ time curves possessed the same four stages as those of the stress-time curves in Fig 3. The results, in terms of damage evolution process, are discussed as follows:

1. Crack closure stage (0*–σ*_*cc*_): Due to small loading and initial microcrack closure, the numerical difference and spatial concentration were negligible in *γ*_xy_, and the strain-field heterogeneity in rock specimens was not evident; therefore, *δ*_*nor*_ and *C*_*v*_ were small and exhibited a relatively constant trend at the early stage (0–186.5 s, 0–158.5 s, 0–152 s for Specimen No. 1, No. 2 and No. 3, respectively). Later at this stage (304.5–420 s, 346–429 s, 302–381.5 s for Specimen No. 1, No. 2 and No. 3, respectively), these were increased with increasing external stress.
2. Linear elastic strain stage (*σ*_*cc*_*–σ*_*ci*_): All parts of the rock specimens maintained a uniform change under loading stress, and the numerical difference and spatial concentration in *γ*_xy_ continued the similar variation trend as that of the previous stage, i.e., *δ*_*nor*_ and *C*_*v*_ increased with a relatively constant rate, while the growth rate increased rapidly at the later stage.
3. Plastic strain stage (*σ*_*ci*_*–σ*_*cd*_ &*σ*_*cd*_*–σ*_*p*_): With microcrack developing in rock specimens, the strain-field heterogeneity in rock specimens was evident, and the larger strain points densely appeared in microcrack developing region, where the macroscopic fracture would form finally. At the early stage, the *C*_*v*_ for the three specimens increased drastically, meanwhile the *δ*_*nor*_ started to grow with a high rate. Later in the stage, the *C*_*v*_ growth slowed down owing to two strain concentration bands (refer ellipse at marker F in Fig 6(B) and 6(C)) for specimens No. 2 and No. 3, while the *C*_*v*_ for specimen No.1 still increased quickly; and due to the numerical difference continuously increasing between the larger and smaller strain point, the *δ*_*nor*_ for the three specimens increased drastically.
4. Post-peak stage (*σ*_*p*_–0): After the peak stress, the macroscopic fracture formed at the larger strain points development area, and *δ*_*nor*_ and *C*_*v*_ reached a maximum. However, the maximum value of *C*_*v*_ for the three specimens had difference, the maximum value of *C*_*v*_ for specimen No.1 equaled to 1 owing to forming one strain concentration band (refer ellipse at marker F in Fig 6(A)), while two strain concentration bands develop with an angle in specimens No. 2 and No. 3 (refer ellipse at marker F in Fig 6(B) and 6(C)), and the maximum value reached to 0.80 and 0.58, respectively. The numerical difference and spatial concentration in strain-field were derived from the crack closure, initiation, penetration, and coalescence in rock specimens. The variation in *δ*_*nor*_ and *C*_*v*_ was closely related to the rock damage; thus, an acceptable agreement was observed among the heterogeneous strain behaviors for the three specimens.

## Discussion

The heterogeneity of strain-field is an outside indication of the microcrack development in rock specimens, and the AE signals are generated because of the elastic energy released during microcrack development [39, 45, 46]. The strain-field heterogeneity shows two features: the numerical difference and spatial concentration close to the sudden failure of the rock specimens with increasing stress, as well as the AE signals increase densely. It is similar in generation mechanism for the heterogeneous strain-field and AE signals, but different in sensitivity to generation mechanism. In this section, the damage precursor born of the *C*_*v*_, *δ*_*nor*_ and AE ring is extracted to investigate the time sequence characteristic.

**Fig 8:**
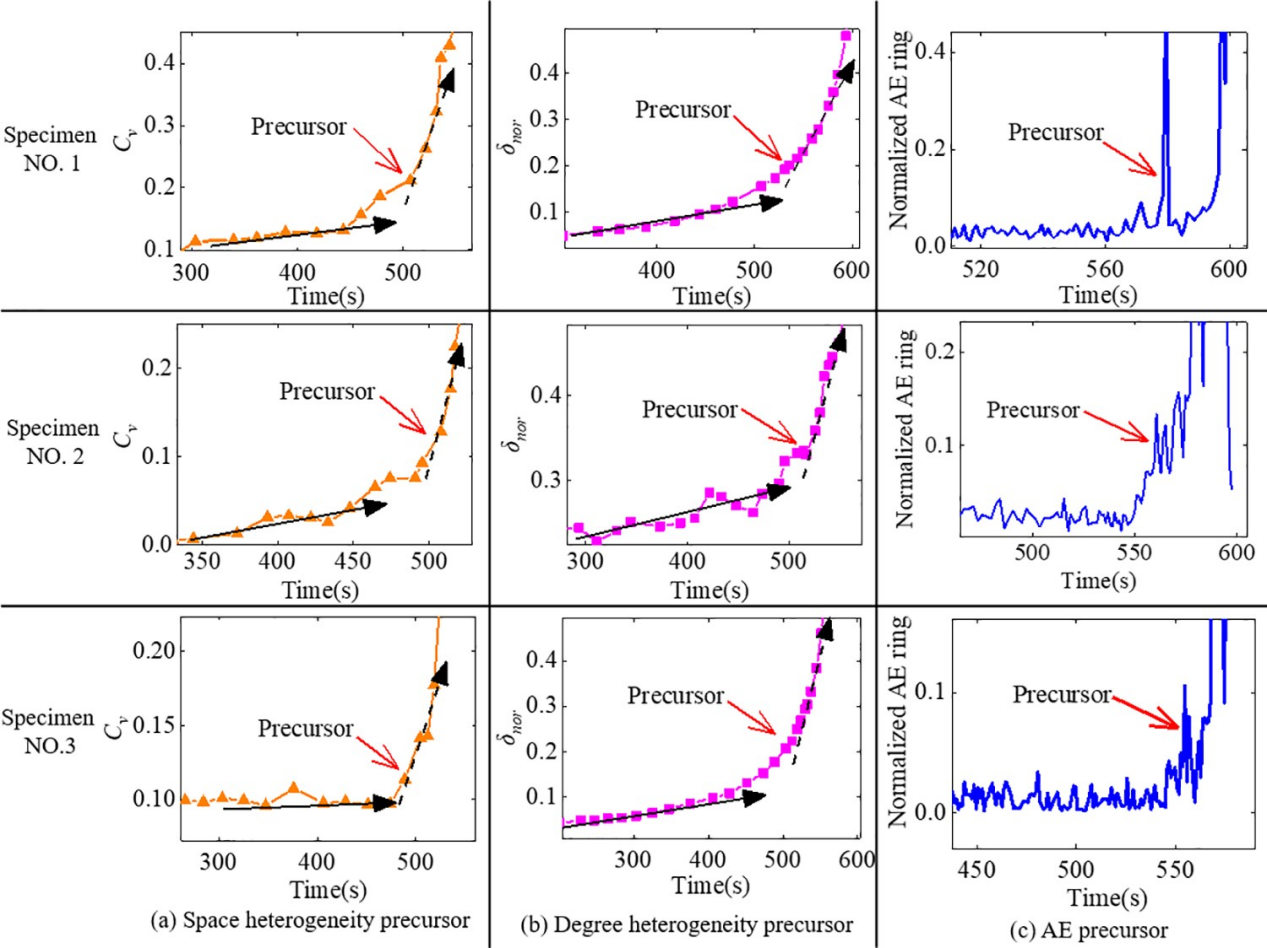
Damage precursors for the three specimens.

As discussed earlier, *C*_*v*_ and *δ*_*nor*_ show four process of transition with loading: a relatively constant trend, growth with a relatively constant rate, drastic increasing trend, and increase with a high rate to maximum value. Considering the drastic increase trend as the initial damage precursor of the rock specimens, the *C*_*v*_ and *δ*_*nor*_ -time curves were amplified locally, and presented in Fig 8(A) and 8(B), respectively. In Fig 8(A) and 8(B), the solid black arrows and the dotted black arrows expressed the low and high development trend of *C*_*v*_ and *δ*_*nor*_ respectively, and the *C*_*v*_ and *δ*_*nor*_ precursors for the three specimens were point out with red arrows. For AE signals, the first unusual rise in AE ring was considered as the initial damage precursor, and the local amplification region were shown in Fig 8(C). It is noteworthy that the pictures in line are for Specimen No. 1, No.2 and No.3 from top to bottom in Fig 8. As shown in Fig 8, three types of damage precursors for the three specimens were obvious, as well as a distinct difference in time. Further, the precursor stress (*σ*_*b*_) and time (*t*_b_), when the damage precursors appeared, were counted in Table 1, meanwhile, the ratio of the precursor stress to the peak stress (*σ*_*b*_/*σ*_*p*_) and the difference between the time of peak stress (*t*_p_) and the precursor time (Δt = *t*_p_-*t*_b_) were calculated. Table 1 reveals the time sequence characteristic among the three types damage precursors. For all three specimens, although *σ*_*b*_ and *t*_b_ of the same precursor type have visible disparity, the difference of *σ*_*b*_/*σ*_*p*_ and Δt is fairly minor. The *σ*_*b*_/*σ*_*p*_ of the *C*_*v*_ precursor for the three specimens is 84.68%, 83.94% and 82.73%, and the difference between maximum and minimum is less than 2%, and the average is 83.78%; the *σ*_*b*_/*σ*_*p*_ of the *δ*_*nor*_ precursor for Specimen No. 1 and No.3 is 95.67%, 95.13%, but for Specimen No. 2 is 84.72%, and the average is 91.84%; also AE precursor has fairly minor difference about the *σ*_*b*_/*σ*_*p*_, the *σ*_*b*_/*σ*_*p*_ is 97.78%, 96.90% and 98.26, with an average of 97.65%. And another conclusion can be drawn: the precursor stress (*σ*_*b*_) of AE precursor is closest to peak stress (*σ*_*p*_), the *σ*_*b*_ of the *δ*_*nor*_ precursor comes second, and the *σ*_*b*_ of the *C*_*v*_ precursor has greatest distance. On the other hand, Δt is the time ahead of the peak stress for three types precursor, the higher the value of Δt, the earlier the damage prediction. The Δt of the *C*_*v*_ precursor for the three specimens is 87 s, 77 s and 85 s, with an average of 83 s, and the Δt of the *δ*_*nor*_ precursor is smaller (33s, 64s and 30s respectively, with an average of 42 s), while the Δt of the AE precursor is smallest, 20s, 25s and 13s respectively, and the average is 19 s. Thus, an acceptable agreement is observed among the three types of damage precursors for the three specimens, that is the time sequence of the three types of damage precursors as follows: *C*_*v*_→*δ*_*nor*_→the AE ring.

**Table 1:** The precursor stress and time for the three specimens.

Specimen	*σ*_p_ (MPa)	*t*_p_ (s)	*C*_*v*_ precursor				*δ*_*nor*_ precursor				AE precursor			
			*σ*_b_ (MPa)	*t*_b_ (s)	*σ*_b_/*σ*_p_ (%)	Δt (s)	*σ*_b_ (MPa)	*t*_b_ (s)	*σ*_b_/*σ*_p_ (%)	Δt (s)	*σ*_b_ (MPa)	*t*_b_ (s)	*σ*_b_/*σ*_p_ (%)	Δt (s)
NO.1	85.5	598.5	72.4	511.5	84.68	87	81.8	565.5	95.67	33	83.6	578.5	97.78	20
NO.2	90.3	584.5	75.8	507.5	83.94	77	76.5	520.5	84.72	64	87.5	559.5	96.90	25
NO.3	86.3	567.0	71.4	482.0	82.73	85	82.1	537.0	95.13	30	84.8	554.0	98.26	13
Average			/	/	83.78	83	/	/	91.84	42	/	/	97.65	19

**Fig 9:**
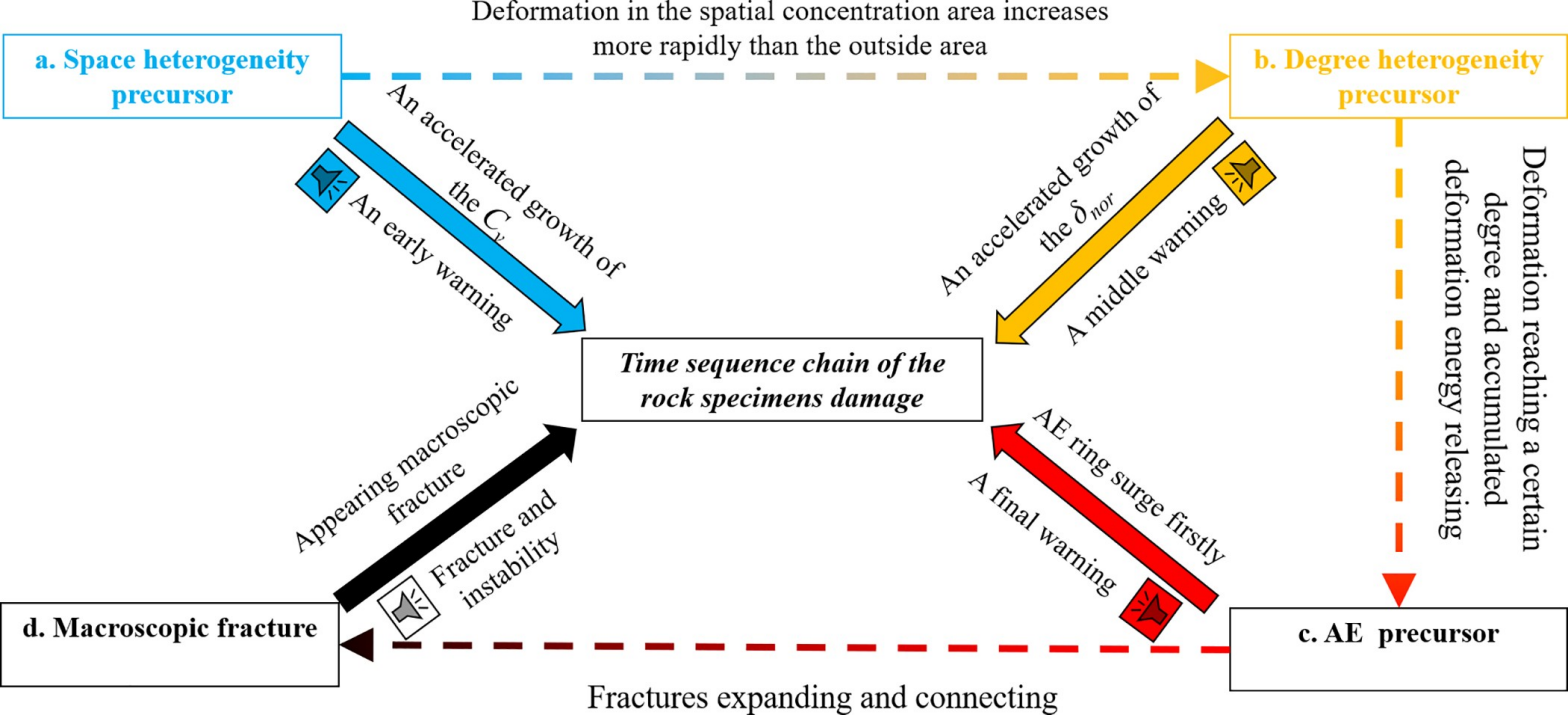
Time sequence chain of the *C*_*v*_ precursor, *δ*_*nor*_ precursor, AE precursor and macroscopic fracture.

The generation mechanism of the three types of damage precursors was closely related to the process of microcrack development. Under external load, the new microcrack developed after the primary microcrack closure. In this process, the heterogeneous strain developed synchronously, where the larger strain points showed spatial concentration first, and the accelerated growth precursor of *C*_*v*_ appeared. Then, the strain in the spatial concentration area increased more rapidly than the outside area, and the *δ*_*nor*_ precursor can be seen. Meanwhile, the strain at the end of the crack reached a certain degree, and the accumulated strain energy would be release. The AE ring surge could be monitored, and the precursor of AE signal appeared. Finally, the fracture started to expand at the end of rock specimens and connected with other fractures to form the macroscopic fracture. This was the reason for the difference in time sequence among the three types of damage precursors. Further, a time sequence chain was prepared by the *C*_*v*_ precursor, *δ*_*nor*_ precursor, AE precursor, and macroscopic fracture, as shown in Fig 9.

As shown in Fig 9, with local strain intensifying in rock specimen, the space heterogeneity precursor appears firstly, and the accelerated growth of *C*_*v*_ can be observed, which predicts that the crack is from initiation to development, it can be used as an early warning of the rock damage. Further, the deformation in local area increases more rapidly than the outside area, and the degree of the strain concentration increases further, and the *δ*_*nor*_ experiences accelerated growth, the degree heterogeneity precursor appears and indicates that the rock damage has developed steadily, which can be used as a middle warning. Subsequently, the AE ring increases sharply near the peak stress which indicates the crack coalescence, and can be considered as a final warning of the rock damage. Finally, the macroscopic fracture appear and cause the rock specimens instability.

## Conclusions

In this study, the time sequence characteristic in heterogeneous strain-field for three sandstone specimens, induced by the external load, was experimentally characterized. Previous studies did not consider quantification of the heterogeneous strain referring to the damage state of the rock specimens. Therefore, quantifying the heterogeneous strain-field and extracting the precursory information of the rock damage are important. In this study, two statistical indicators for the numerical difference and spatial concentration in strain-fields were proposed to investigate the variation in time sequence and express the characteristic damage state. The following conclusions can be drawn:

1. With increasing magnitude of the uniaxial compressive stress, the rock specimens experienced five damage stages characterized by the AE parameters. The variation of the displacement gradient in the displacement-field was from balancing development to heterogeneous variation in damage evolution process. Moreover, the heterogeneous phenomenon was more evident at the top and bottom of the rock specimens entering the plastic strain stage. While in strain-field, the strain value was small and uniform at low levels of stress, then demonstrated a polarization phenomenon with increasing loading. For instance, at larger strain points, it converged and increased rapidly in magnitude; the converged regions could represent latent damage initiation in the rock specimens. Hence, the strain-field heterogeneity showed two features: numerical difference and spatial concentration.
2. In quantifying the strain-field heterogeneity of the rock specimens, *δ*_*nor*_ and *C*_*v*_ were proposed to characterize the numerical difference and spatial concentration, respectively. Further, *δ*_*nor*_ and *C*_*v*_ in strain-field experienced four process of transition with loading: a relatively constant trend, growth with a relatively constant rate, drastically increasing trend, and an increase with a high rate to maximum value; it was the outside indication of the microcrack development and consistent with the damage evolution process of the rock specimens.
3. The trend of drastic increase in *δ*_*nor*_ and *C*_*v*_ indicated the microcrack initiation and coalescence, respectively, with the first rapid increase in AE ring. A time sequence chain about the damage precursor in rock specimens was prepared, and showed that the priority of their appearance in the damage evolution process of the rock specimens was as follows: *C*_*v*_→*δ*_*nor*_→AE ring. Three types of damage precursors for the rock specimens were similar in generation mechanism but differed in sensitivity to microcrack development; these could be used as damage warning for the varying magnitude of the external load.

## Supporting information

S1 Data (ZIP)
